# Design and analysis of HSC-BPPV diagnostic maneuver based on virtual simulation

**DOI:** 10.3389/fneur.2023.1132343

**Published:** 2023-02-17

**Authors:** Yanjun Li, Xiaokai Yang

**Affiliations:** Department of Research Center, Third Affiliated Hospital of Shanghai University, Wenzhou Third Clinical Institute Affiliated to Wenzhou Medical University, Wenzhou People's Hospital, Wenzhou, China

**Keywords:** BPPV, virtual simulation, supine roll test, horizontal semicircular canal, diagnostic maneuver

## Abstract

**Background:**

The preferred supine roll test for the diagnosis of horizontal semicircular canal BPPV has several disadvantages, including difficulty in locating the affected ear, inconsistent nystagmus performance on repeated testing, and lack of a typical latency period, resulting in insensitive diagnosis.

**Objectives:**

To investigate novel diagnostic techniques with more scientific design, more accessible application, and better diagnostic sensitivity and specificity.

**Materials and methods:**

Based on clinical microscopic CT data, we created a virtual simulation model of BPPV using Unity software. The physical simulation of the traditional supine roll test was performed to observe and analyse the movement of the otoliths, whose initial position was the typical stable position. In addition, the normal vectors of the plane and crista ampullaris of the horizontal semicircular canal were measured using 3D Slicer software. Based on this, we analyzed the critical steps for designing diagnostic maneuvers for BPPV in the horizontal semicircular canal. For a more accurate diagnosis of horizontal semicircular canal BPPV, it is critical to rotate the horizontal semicircular canal to be parallel to gravity. It is also necessary to move the otolith by swinging the head. As a result, we developed two diagnostic maneuvers: the 60° roll test and the prone roll test. We also performed simulations to observe otolith movement and predict nystagmus performance.

**Conclusions:**

The 60° roll test and the prone roll test can complement the supine roll test. Compared to the supine roll test, they not only effectively differentiate canalolithiasis from cupulolithiasis, but also make it easier to determine the position of the otoliths, and the characteristics of the nystagmus are more pronounced. Significant diagnostic features have significant potential benefits for home and telemedicine.

## 1. Introduction

**Figure 1:**
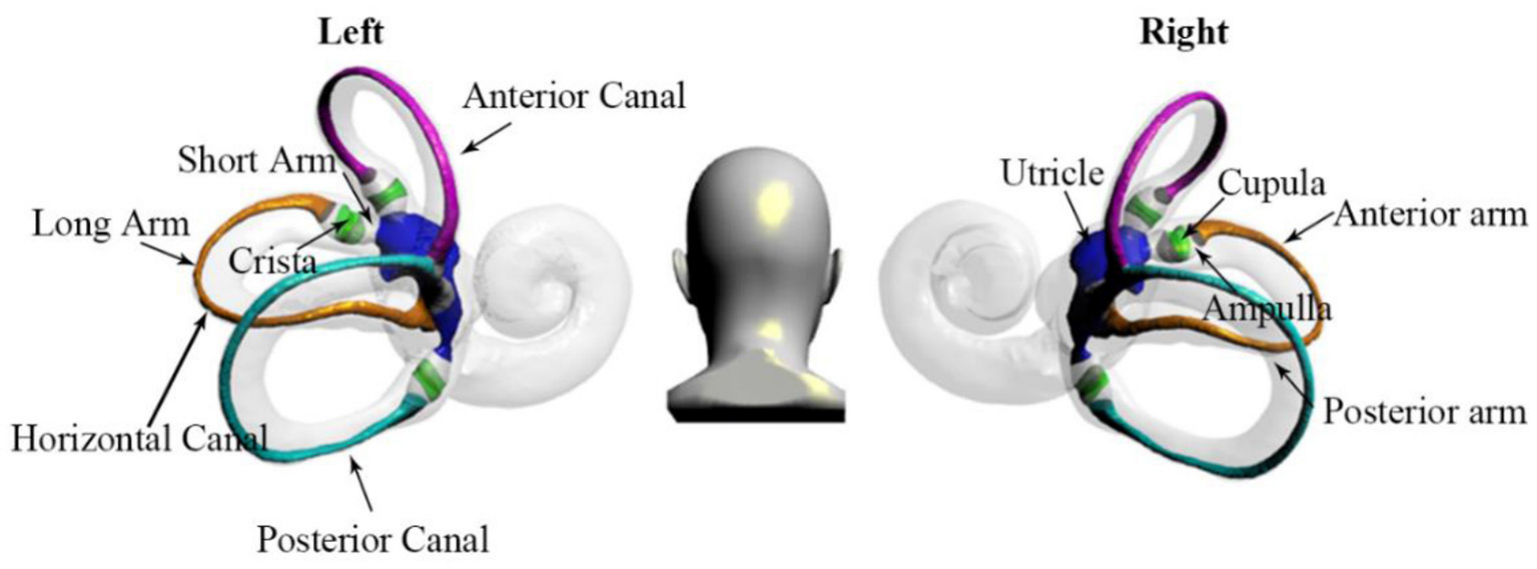
The structure of the semicircular canal shows the anterior, horizontal, and posterior canals. By using the crista as a boundary, the horizontal semicircular canal is divided into short and long arms. In addition, the long arm is divided into anterior and posterior parts.

The most common vertigo disorder, known as benign paroxysmal positional vertigo (BPPV), is characterized by recurrent episodes of positional vertigo and nystagmus induced by changes in the position of the head relative to gravity (1, 2). The pathophysiology is caused by free-floating otoliths adhering to the cupula (cupulolithiasis) or moving freely in the semicircular canal (cupulolithiasis) (3–5). The schematic diagram of the semicircular canal structure is shown in Figure 1. Posterior semicircular canal BPPV is the most common, succeeded by horizontal semicircular canal BPPV (HSC-BPPV), with an incidence ranging from 5 to 30% (6, 7).

According to Ewald's first law, stimulation of the semicircular canal causes a movement of the eyes in the plane of the stimulated canal (8). Hence, specific diagnostic positional maneuvers can be used to induce vertigo and nystagmus in order to identify the affected semicircular canal by analyzing the characteristics of the nystagmus. The otolith can be moved to the utricle by correct repositioning (9).

In clinical practice, HSC-BPPV is usually diagnosed and located by the supine roll test based on the intensity, direction, and duration of nystagmus (10). In most cases, the otolith is located in the long arm of the semicircular canal, and geotropic nystagmus is evoked when the head is turned to one side during the supine roll test (11). Moreover, it is characterized by intense horizontal nystagmus beating toward the undermost (affected) ear. Less frequently, the otolith is located near the ampulla of the semicircular canal or adhering to the cupula, and apogeotropic nystagmus is evoked when the head is turned to one side while performing the supine roll test. Moreover, it is characterized by intense horizontal nystagmus beating toward the uppermost (affected) ear. Apogeotropic nystagmus induced by the supine roll test is considered to be cupulolithiasis. It is more intense when the healthy side is undermost.

However, there are still many problems with the clinical application of the supine roll test (12). First, it is difficult to identify the affected ear because the intensity of the nystagmus induced by head rotation on both sides may not be easily distinguishable or may change with repeated testing. Second, there is no typical latency period. Nystagmus elicited by the supine roll test has no latency or a short latency period. Third, the position of the otolith may be changed due to the operation of the supine roll test, which may lead to inconsistent nystagmus in repeated diagnostic tests and affect the judgment of the doctor.

Therefore, it is necessary to redesign and scientifically demonstrate HSC-BPPV diagnostic maneuvers. The BPPV simulation model can dynamically and intuitively observe the otolith movement triggered by the diagnostic maneuver (13–15). Furthermore, it is an excellent auxiliary means for designing and analyzing BPPV diagnosis and therapy maneuvers. However, the previous simulation models adopted a simplified membrane semicircular canal model which ignored the critical factors of different hydrodynamic effects caused by the local enlargement of the crista ampullaris, and thus could not accurately simulate the actual situation (16, 17).

Here, we performed a physical simulation and analysis of the horizontal semicircular canal diagnostic maneuver based on a previously established BPPV simulation model (15, 18–20). To facilitate the design of the BPPV diagnostic test, we measured the spatial orientation of the membranous semicircular canal and the crista ampullaris (21). In addition, the physical simulation and analysis of the traditional supine roll test were carried out with the BPPV simulation model. The conclusion was consistent with the previous research. Furthermore, the 60° roll and prone roll test were designed to supplement the diagnosis of HSC-BPPV, making the diagnosis more convenient and accurate. It is conducive to self-diagnosis and remote diagnosis of patients at home.

## 2. Methods

### 2.1. To obtain the membrane labyrinth model

Clinical imaging data can capture the bone labyrinth structure but not or only partially reveal the membrane labyrinth structure. The microscopic CT data can show the structure of the bone labyrinthine and membrane labyrinthine, but the spatial orientation information of the inner ear is lacking, so it is necessary to indirectly determine the spatial direction through calibration.

Bone and membrane labyrinth models were extracted from clinical micro-CT images to obtain semicircular canal models that approximate the anatomy. The spatial orientation of the model was calibrated by establishing a standard three-dimensional coordinate system (22). There are individual differences in the spatial orientation of the inner ear. Therefore, this study established a standard three-dimensional coordinate system based on reconstructed magnetic resonance imaging (MRI) of 55 normal human inner ears to obtain a representative model of a membranous labyrinth (21). Firstly, bilateral inner ear and eyeball models were obtained by MRI image segmentation to generate statistical shape models, and the average model was derived as the standard model. Then, the standard three-dimensional space coordinate system was established with the total foot bifurcating point of the semicircular canal and the lower edge of the eyeball as the horizontal plane. Finally, the bone labyrinth models extracted from microscopic CT examination data were calibrated with the standard model. And then, the membrane labyrinth models were subjected to three-dimensional spatial transformation according to the calibration results to establish the spatial direction (18).

### 2.2. To build a BPPV simulation model

The BPPV virtual simulation model is based on Unity 3D software (version 2020.3) and the built-in NVIDIA Physx physics engine developed using browser and server architecture (20). The key steps are as follows:

#### 2.2.1. Parameter settings

The radius of otoliths ranges from 0.5 to 15 nm, with an average radius of 7.5 nm. The density of the otolith is 2.71 *g/cm*^3^, the density of the endolymph is 1 *g/cm*^3^, and the buoyancy is set at 3.62 *m/s*^2^ (20).

#### 2.2.2. Otolith initial position setting

The horizontal sitting position was selected as the semicircular canal space position for setting the initial position of the otolith. Otoliths were placed in each major position of the horizontal canal. After starting the simulation, the otolith will naturally settle to the lowest position under the action of gravity.

### 2.3. Operation of the supine roll test and the principle of predicting nystagmus characteristics

The simulation sequence of the horizontal roll test was from the flat sitting position to the supine position with the head raised at 25°, turn over 90° on the right side first, then return to the flat supine position, and then turn over 90° on the left side. Otolith movement at different positions of the horizontal semicircular canal was observed at each step, and corresponding nystagmus characteristics were predicted according to vestibular physiological principles. According to Ewald's second law, when the crista ampullaris of HSC is stimulated, the endolymph toward the ampulla produces strong excitatory nystagmus, while the endolymph away from the ampulla produces weak inhibitory nystagmus (23, 24). Since the otolith adhered to the cupula and increased the specific gravity of the crista, the stimulation effect of gravity on the cilia of the ampullary crest hair cells was more lasting when moving. Hence, the intensity and duration of nystagmus induced by the crest stones were more significant and prolonged, usually more than 1 min.

### 2.4. Design of diagnostic tests and analysis of critical steps

To make the starting position of the otolith movement consistent in the diagnostic test, the horizontal semicircular canal can be rotated parallel to the direction of gravity so that the otolith in the horizontal semicircular canal slides to the same position under the action of gravity.

**Figure 2:**
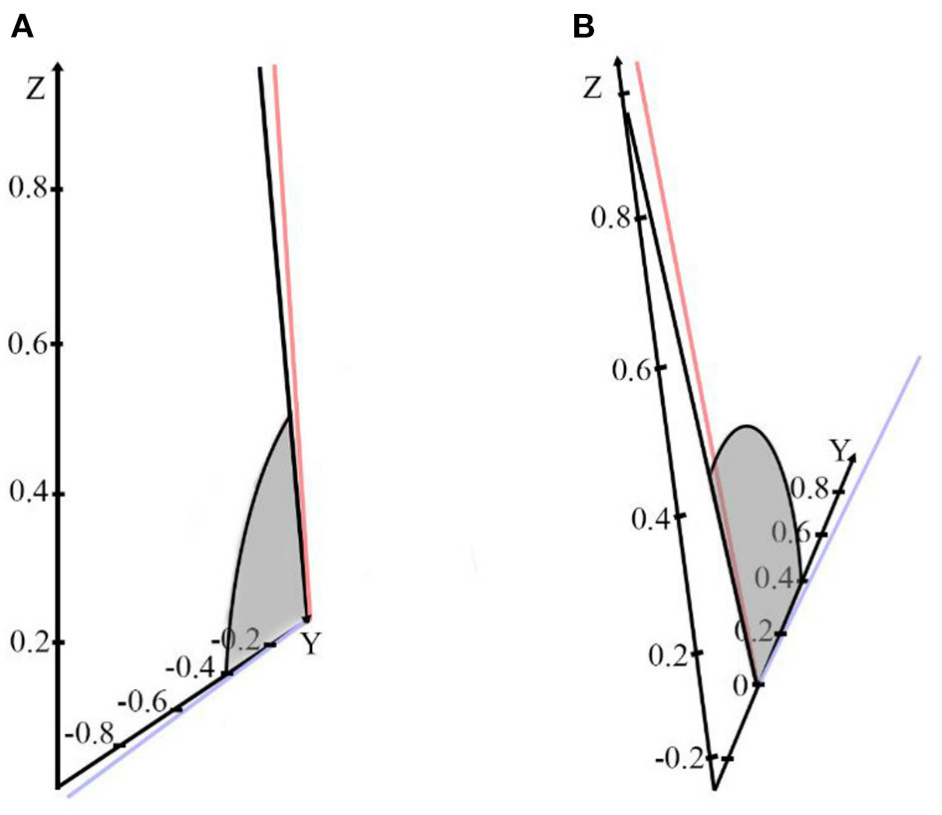
**(A)** The horizontal semicircular canal normal vector is rotated 73.8° clockwise around the X-axis to the XY plane. **(B)** The normal vector of the horizontal semicircular canal is rotated 106.17° counterclockwise around the X-axis to the XY plane.

Specify the forward direction of the coordinate axis as z-axis up, Y-axis inward, and X-axis to the right. The normal vector of the horizontal semicircular canal was rotated to the XY plane by calculating, and the results were as follows: (1) Rotate 73.8° clockwise around the X axis to the XY plane, as shown in Figure 2A; (2) Rotate 106.17° counterclockwise around the X-axis to the XY plane, as shown in Figure 2B. These results suggest that the left or right decubitus position can make the crista ampullaris of the horizontal semicircular canal close to parallel to the direction of gravity.

## 3. Results

We use the simulation model to set the otolith's initial position and study the otolith's stable settlement position. In addition, the physical simulation and analysis of the traditional supine roll test were carried out and based on this, the 60° roll test and the prone roll test were designed.

### 3.1. Position of otoliths settlement in a sitting position

Under the action of gravity, the otolith often sinks to the local lowest position of the horizontal semicircular canal.

**Figure 3:**
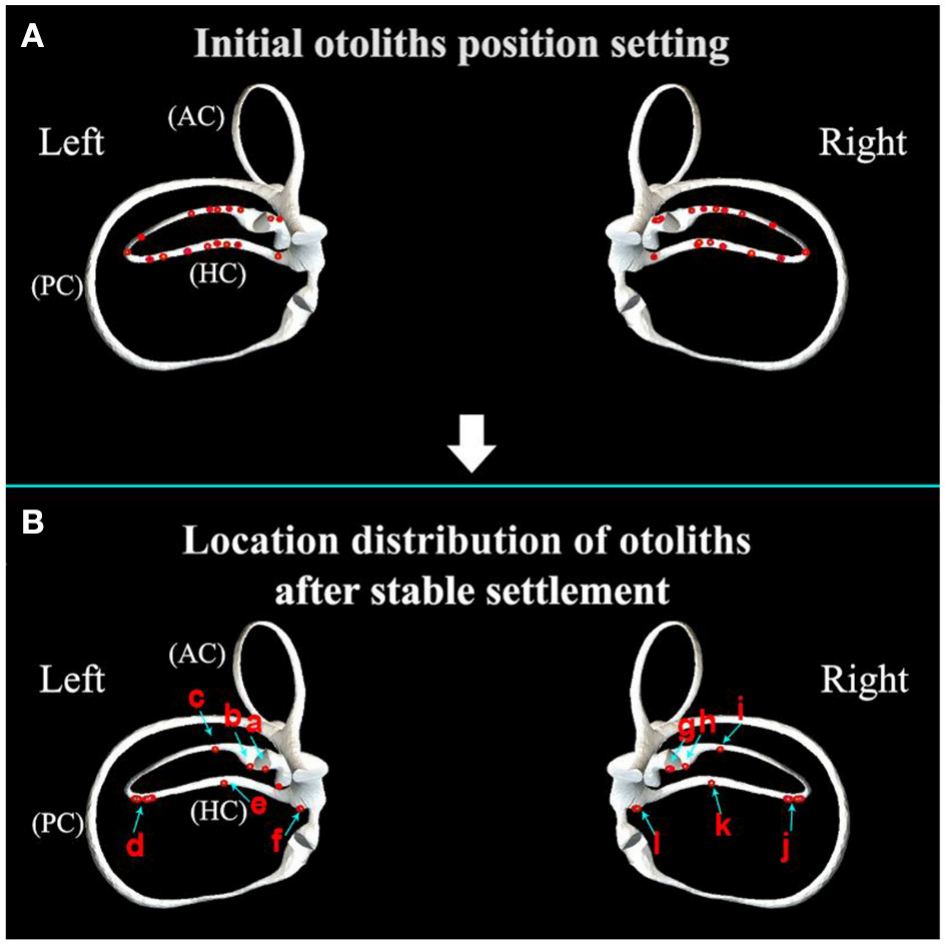
**(A)** Initial otoliths position setting in the horizontal semicircular canal; **(B)** location distribution of otoliths after stable settlement. (a) The side of the short arm (the ampulla near the utricle); (b) the ampulla of the long arm; (c) the anterior arm of the long arm; (d) the posterior arm of the long arm; (e) the posterior arm is close to the opening of the utricle; (f) utricle opening of the posterior arm.

As shown in Figure 3A and Supplementary Video S1, the initial position of otoliths was set on the horizontal semicircular canal model in the horizontal sitting position. The position of otoliths after the stable settlement is mainly distributed in six parts, as shown in Figure 3B. Namely, point a is located in the ampulla on the short arm side, and point b is located in the ampulla on the long arm side. Point c is positioned in the anterior arm of the long arm, near the ampulla. In the posterior arm of the long arm, point d is found. Point e is near the utricular opening, and point f is at the opening of the utricle. Therefore, we selected three specific positions a, b, and d, as the initial locations for subsequent simulation of horizontal semicircular canal diagnostic maneuvers.

### 3.2. Initial location selection of otoliths

**Figure 4:**
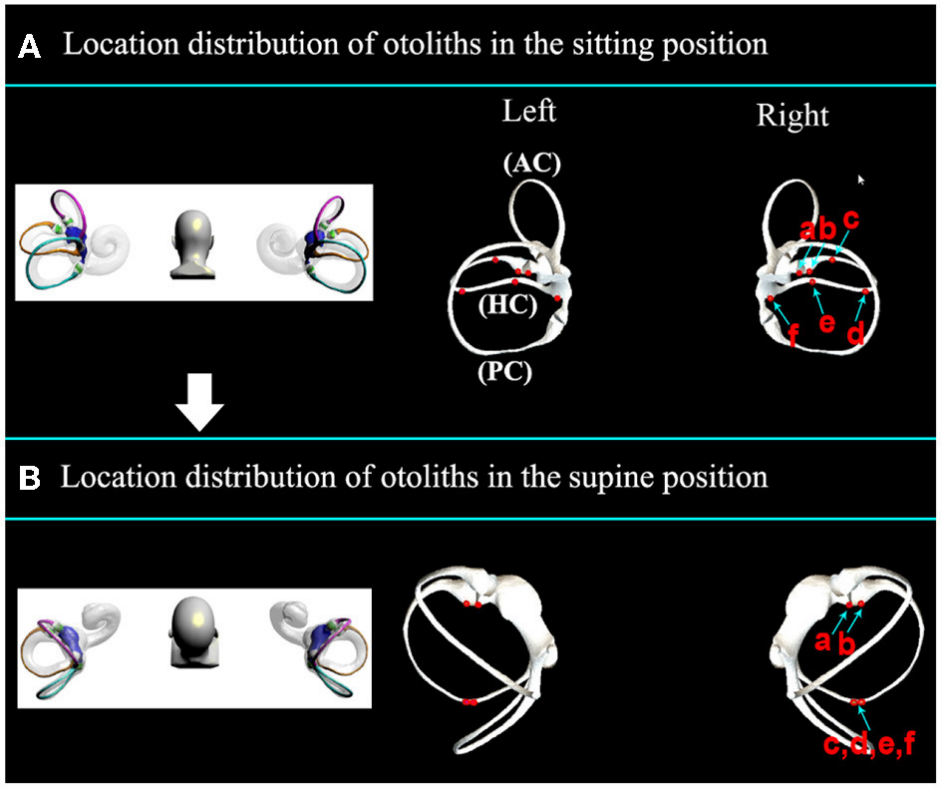
**(A)** Distribution of otoliths in the horizontal semicircular canal in the sitting position. Otolith a is located on the short arm side (ampulla near the utricle); otolith b is located in the ampulla of the long arm; otolith c is located in the anterior of the long arm; otolith d is located in the posterior arm of the long arm; otolith e is located in the posterior arm near the opening of the utricle; otolith f is located at the opening of the utricle of the posterior arm. **(B)** The distribution of otoliths in the horizontal semicircular canal after stable settlement from the sitting position to the supine position.

The positional distribution of otoliths in the sitting position is shown in Figure 4A. When changing from the sitting position to the supine position, otoliths moved under the action of gravity, and the stable distribution of otoliths is shown in Figure 4B and Supplementary Video S2. Otoliths in the right and left horizontal semicircular canal followed the same trajectory. On the right side, otoliths c and d moved toward the ampulla, and the affected side was in the direction of the nystagmus. Otoliths e and f moved away from the ampulla, and the healthy side was the direction the nystagmus faced. There was no nystagmus produced by otoliths a and b in the ampulla. On the cupula, otoliths were adhered to and did not induce nystagmus. Otoliths c, d, e, and f were all concentrated together after stabilization in the supine position. Therefore, the stabilized position of the posterior arm was chosen as the starting position for the prone position to simplify the subsequent description.

### 3.3. Physical simulation and otolith motion observation of the supine roll test

**Figure 5:**
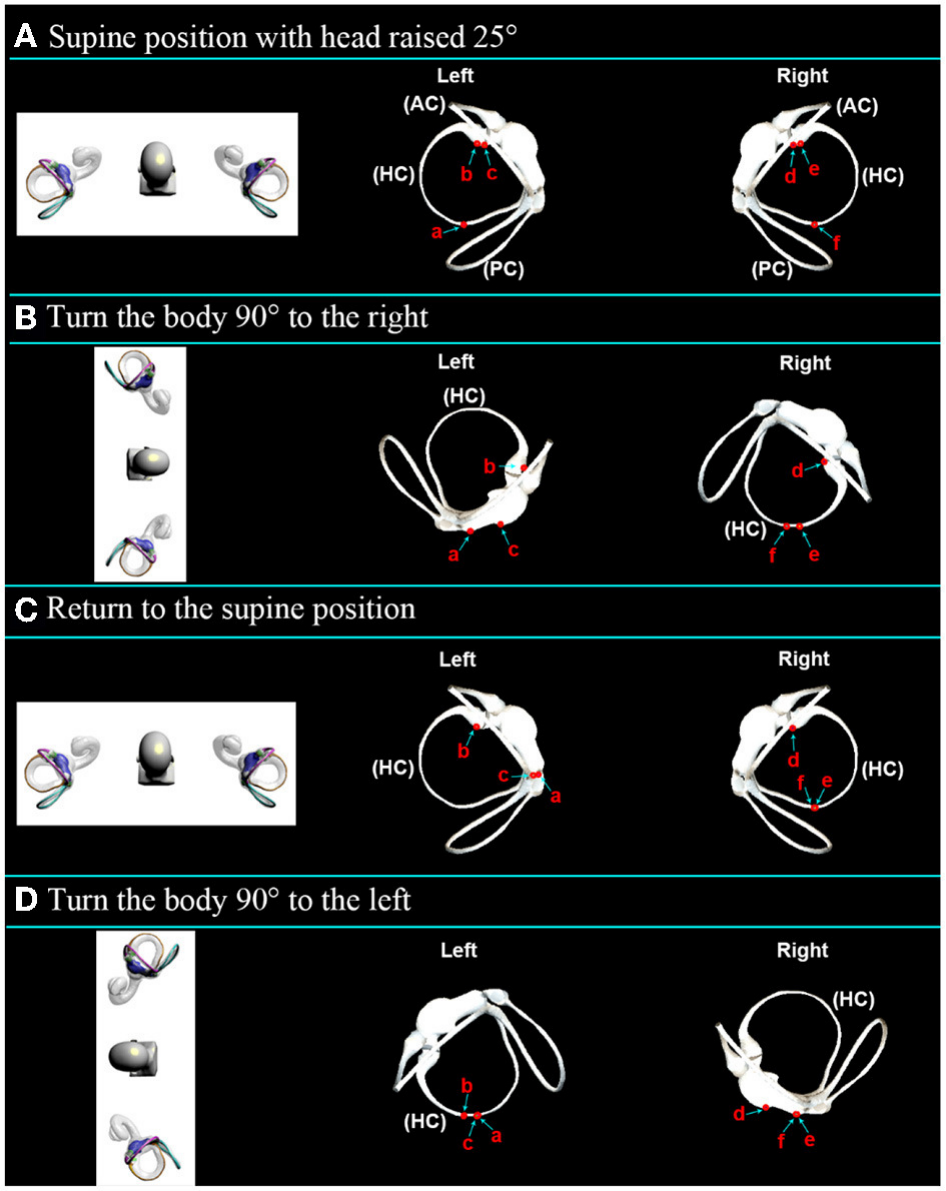
Movement of the otoliths in the horizontal semicircular canal in the supine roll test. **(A)** The stable position of otoliths in the supine position with the head raised 25°. **(B)** The stable position of otoliths after turning to the right at 90°. **(C)** The stable position of otoliths after returning to the supine position. **(D)** The stable position of otoliths after turning 90° to the left.

Turning left or right first in the supine roll test affects evoked nystagmus performance. Here, it was set as the inspection order: first turn right, then left. As shown in Supplementary Video S3, the supine roll test simulation was carried out after the initial position was set. The observed otolith movement and the predicted nystagmus characteristics are shown in Figure 5 and Table 1:

(1) Step A: As shown in Figure 5A, the supine position with the head is raised 25° to make the horizontal semicircular canal vertical with respect to gravity.
(2) Step B: Turn the body 90° to the right, as shown in Figure 5B. On the left side, the otolith a on the side of the long arm was far away from the ampulla and entered the utricle, inducing geotropic nystagmus. The ampullary otolith b on the long arm side moved to the bottom of the crista at a short distance. The otolith c on the side of the short arm moved toward the ampulla and entered the utricle, inducing apogeotropic nystagmus. The cupulolithiasis evoked apogeotropic nystagmus. On the right side, the otolith f on the side of the long arm moved toward the ampulla to induce geotropic nystagmus. The ampullary otolith e on the side of the long arm moved away from the ampullary, inducing apogeotropic nystagmus. The otolith d on the top of the crista induced apogeotropic nystagmus. Apogeotropic nystagmus was induced by cupulolithiasis.
(3) Step C: Return to the supine position, as shown in Figure 5C. On the left, otoliths a and c moved in the utricle. The ampullary otolith b on the side of the long arm moved a short distance to the top of the crista. The cupulolithiasis did not induce nystagmus. On the right, otoliths e and f on the long arm side moved away from the ampulla and induced nystagmus horizontally toward the left side. The position of stone d on the side of the short arm did not change. The cupulolithiasis did not induce nystagmus.
(4) Step D: Turn the body 90° to the left, as shown in Figure 5D. On the left side, otoliths a and c on the long arm side moved out of the utricle toward the ampulla and induced geotropic nystagmus. Otolith b on the short arm side moved away from the ampulla to induce apogeotropic nystagmus. The cupulolithiasis induced apogeotropic nystagmus. On the right side, otoliths e and f on the long arm side were far away from the ampulla and entered the utricle to induce geotropic nystagmus. Otolith d on the short arm side entered the utricle toward the ampulla and induced apogeotropic nystagmus. Apogeotropic nystagmus was induced by cupulolithiasis.

**Table 1:** Otolith motion and nystagmus speculation in the supine roll test.

**Otolith**	**Step A**	**Step B**	**Step C**	**Step D**
Otolith a was located on the PA of the LA	AFM/ →	AFM/weak ↓	Movement within the utricle/–	Movement in the utricle or out of the utricle to the ampulla/– or strong ↓
Otolith b was located on the ampulla of the LA	The TC on the LA/–	Move to the BC/–	Move to the TC/–	AFM/weak ↑
Otolith c was located on the ampulla of the SA	The TC on the SA/–	APM into the utricle/strong ↑	Movement within the utricle/–	Movement in the utricle or out of the utricle to the ampulla/– or strong ↓
Otolith adhered to the cupula of the left ear	Adhesion state/–	Adhesion state/strong ↑	Adhesion state/–	Adhesion state/weak ↑
Otolith d was located on the ampulla of the SA	The TC on the SA/–	The TC on the SA/weak ↑	The TC on the SA/–	APM into the utricle/strong ↑
Otolith e was located on the ampulla of the LA	The TC on the LA/–	AFM/weak ↑	AFM/←	AFM into the utricle/weak ↓
Otolith f was located on the PA of the LA	AFM/ →	APM/strong ↓	AFM/←	AFM into the utricle/weak ↓
Otolith adhered to the cupula of the right ear	Adhesion state/–	Adhesion state/weak ↑	Adhesion state/–	Adhesion state/strong ↑

PA, Posterior arm; LA, Long arm; SA, Short arm; TC, Top of the crista; BC, Bottom of the crista; AFM, Ampullofugal movement; APM, Ampullopetal movement; –, Ineffective nystagmus; ↓, Geotropic nystagmus; ↑, Apogeotropic nystagmus; ←, Nystagmus horizontally toward the left side; →, Nystagmus horizontally toward the right side.

In general, the supine roll test can be used to diagnose the following according to the characteristics of nystagmus:

(1) If both steps B and D induce geotropic nystagmus, the otolith is considered to be located in the long arm of the horizontal canal, including the anterior arm near the ampulla, the posterior arm, and the opening of the utricle, and the opening of the utricle. Moreover, nystagmus is more intense when moving toward the affected ear. Further localization can be done based on the intensity and direction of nystagmus from sitting to supine. First, the otolith at or near the opening of the utricle elicits excitatory stimuli with nystagmus toward the side of the affected ear. And nystagmus is stronger at the opening of the utricle. Secondly, the otolith near the ampulla or in the posterior arm caused nystagmus toward the unaffected side. Nystagmus is stronger near the ampulla.
(2) Apogeotropic nystagmus for more than 1 min in steps B and D was considered cupulolithiasis. If the duration was <1 min, the otolith was considered in the right ear's short arm. The nystagmus was more intense when the head was turned to the healthy side.
(3) Step B or D induces apogeotropic nystagmus lasting <1 min, considering that the otolith was located in the ampulla of the semicircular canal. First, step B did not induce nystagmus, while step B could induce apogeotropic nystagmus. The otolith was considered to be located in the long arm side of the left ear. Second, when step B induced weak apogeotropic nystagmus and step D induced weak geotropic nystagmus, the otolith was considered to be located in the long arm side of the right ear. Third, when step B induced strong apogeotropic nystagmus and step D induced no nystagmus or strong geotropic nystagmus, the otolith was considered to be located in the short arm side of the left ear. This is because the otolith in the short arm may re-enter the long arm by opening the utricle after returning to the utricle.
(4) In particular, if the supine roll test successfully resets the otolith of the cupula on the long arm side, it means that the otolith is detached from the cupula into the long arm, which is manifested as apogeotropic nystagmus in the decubitus position on the affected side and geotropic nystagmus in the decubitus position on the healthy side.
(5) If strong nystagmus occurs in step C, consider that the otolith re-enters the horizontal semicircular canal, which is an irritating stimulus, and the direction of the nystagmus refers to the affected side, and it is considered that the otolith is located on the long arm side of the left ear. Usually, there is also significant nystagmus from the sitting position to the supine position. In addition, the canal otolith of the left long arm side can enter the utricle in the right lateral decubitus position, which can significantly affect the diagnosis. If the otolith that returns to the utricle in step C re-enters the horizontal semicircular canal, step D will also show strong nystagmus, with the more strong nystagmus on the affected side. If the otolith from step C returns to the utricle with little or no re-entry into the horizontal semicircular canal, step D may show weak or no nystagmus, with the more strong nystagmus on the healthy side.

The above is the ideal diagnosis, but there are still many problems in clinical practice. For example, to distinguish BPPV from other diseases that cause vertigo and nystagmus, repeated diagnosis is often required to confirm BPPV. However, rolling the body to the healthy side in the supine roll test can cause otoliths to reset, thus affecting the sensitivity of the diagnostic test.

### 3.4. Principles of diagnostic test design

Based on the analysis of the traditional horizontal roll test, it is necessary to design a new diagnostic maneuver for the horizontal semicircular canal that meets the following conditions: 1() it does not cause free otoliths in the utricle to enter the semicircular canal; (2) cupula otoliths, short-arm lateral canal otoliths, and long-arm lateral canal otoliths have different nystagmus performance; (3) repeated tests have consistent nystagmus manifestations and do not cause otolith repositioning; (4) the induced nystagmus has a long latency period.

A new horizontal semicircular canal BPPV diagnostic test can be designed as follows: (1) Rotate the horizontal semicircular canal parallel to the direction of gravity, i.e., 16° head up in the supine position or 16° head down in the prone position; (2) Induce otolith movement by swinging the head from side to side. The appropriate swing angle is determined by observing the otolith movement in the left and right head swings.

### 3.5. Modified diagnostic test and observation of otolith motion

#### 3.5.1. The 60° head roll test

**Figure 6:**
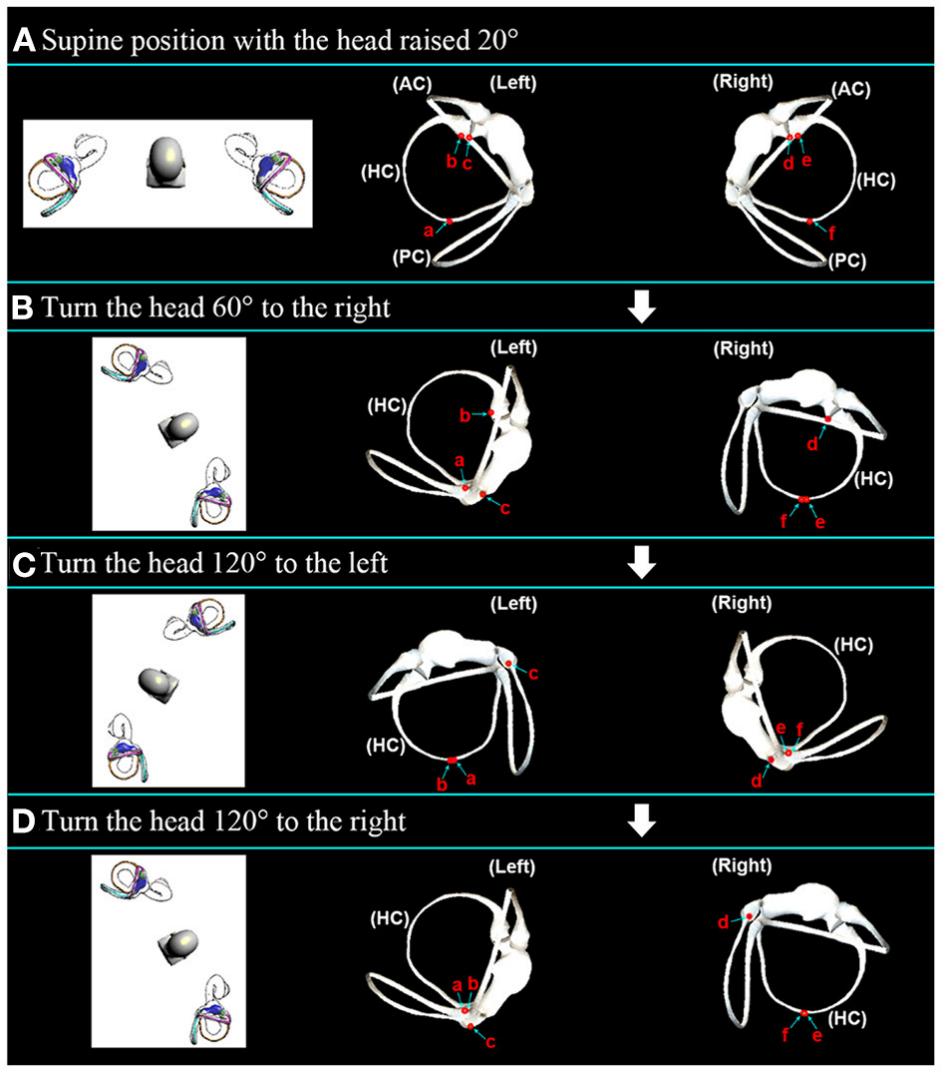
The movement of the otoliths in the horizontal semicircular canal during the 60° roll test. **(A)** The stable position of the otoliths in the supine position with the head elevated 20°. **(B)** The stable position of the otoliths after 60° right rotation. **(C)** The stable position of the otoliths after turning 120° to the left. **(D)** The stable position of the otoliths after 120° right rotation.

The 60° roll test was designed from a flat sitting position to a supine position raised 20°, then the head turned 60° to the right, then 120° to the left, and finally 120° to the right. The 60° roll test simulation was carried out and shown in Supplementary Video S4. The simulation results are shown in Figure 6 and Table 2, and the related nystagmus induced by otolith movement is speculated as follows:

(1) Step A: Supine position with the head raised 20°, as shown in Figure 6A.
(2) Step B: Turn the head 60° to the right, as shown in Figure 6B. On the left side, otolith a on the long arm side moved away from the ampulla to induce geotropic nystagmus; otolith b was located at the top of the crista to induce apogeotropic nystagmus; otolith c on the short arm side moved toward the ampulla and entered the utricle to induce apogeotropic nystagmus; the cupulolithiasis induced apogeotropic nystagmus. On the right side, otolith e in the ampulla of the side of the long arm moved away from the ampulla to induce apogeotropic nystagmus; otolith f on the posterior arm of the long arm moved toward the ampulla to induce geotropic nystagmus; otolith d on the short arm side was located at the top of the crista and induced apogeotropic nystagmus; the cupulolithiasis induced apogeotropic nystagmus.
(3) Step C. Turn the head 120° to the left, as shown in Figure 6C. On the left side, otolith a on the long arm side moved toward the ampulla to induce geotropic nystagmus; the ampullary otolith b on the side of the long arm moved away from the ampulla to induce apogeotropic nystagmus; otolith c on the long arm side moved in utricle or re-entered the short arm; the cristae cap stones induced apogeotropic nystagmus. On the right side, otolith e and f on the long arm side moved away from the ampulla to cause geotropic nystagmus; otolith d moved toward the ampulla into the utricle and induced apogeotropic nystagmus; the cupulolithiasis induced apogeotropic nystagmus.
(4) Step D: Turn the head 120° to the right, as shown in Figure 6D. On the left side, otolith a and b on the long arm side moved away from the ampulla to induce geotropic nystagmus; the location of otolith c did not change significantly; the cupulolithiasis induced apogeotropic nystagmus. On the right side, otolith e and f on the long arm moved toward the ampulla to induce geotropic nystagmus; otolith d moved in utricle or re-entered the short arm; the cupulolithiasis induced apogeotropic nystagmus.

**Table 2:** Otolith motion and nystagmus speculation in the 60° head roll test.

**Otolith**	**Step A**	**Step B**	**Step C**	**Step D**
Otolith a was located on the PA of the LA	AFM/ →	AFM/weak ↓	APM/strong ↓	AFM/weak ↓
Otolith b was located on the ampulla of the LA	The TC on the LA/–	The TC on the LA/strong ↑	AFM/weak ↑	AFM/weak ↓
Otolith c was located on the ampulla of the SA	The TC on the SA/–	APM into the utricle/strong ↑	Movement in the utricle or re-entering the SA/–	Movement in the utricle or re-entering the SA/–
Otolith adhered to the cupula of the left ear	Adhesion state/–	Adhesion state/strong ↑	Adhesion state/weak ↑	Adhesion state/strong ↑
Otolith d was located on the ampulla of the SA	The TC on the SA/–	The TC on the SA/weak ↑	APM into the utricle/strong ↑	Movement in the utricle or re-entering the SA/–
Otolith e was located on the ampulla of the LA	The TC on the LA/–	AFM/weak ↑	AFM/weak ↓	APM/strong ↓
Otolith f was located on the PA of the LA	AFM/←	APM/strong ↓	AFM/weak ↓	APM/strong ↓
Otolith adhered to the cupula of the right ear	Adhesion state/–	Adhesion state/weak ↑	Adhesion state/strong ↑	Adhesion state/weak ↑

–, Ineffective nystagmus; ↓, Geotropic nystagmus; ↑, Apogeotropic nystagmus; ←, Nystagmus horizontally toward the left side; →, Nystagmus horizontally toward the right side.

The following conclusions for nystagmus induced by the 60° roll test can determine the otolith position:

(1) If steps B, C, and D all induce apogeotropic nystagmus, it is considered to be cupulolithiasis, and the nystagmus on the unaffected side is stronger.
(2) If steps B, C, and D all induce geotropic nystagmus, the otolith is considered to be located in the long arm of the horizontal canal, and the nystagmus on the affected side is stronger.
(3) If steps B and D induce apogeotropic nystagmus and geotropic nystagmus, respectively, it is considered that the otolith is located in the ampulla of the long arm side. Further, in step C, apogeotropic nystagmus is induced, and the affected side is the left ear, and geotropic nystagmus is induced, and the affected side is the right ear.
(4) Otolith on the short arm side will enter the utricle.

This test has the following advantages:

(1) Easy to operate. Turning the head to the left and right at 60° can be done easily without turning over and can also avoid cervical spine injury.
(2) Increased sensitivity. The 120° head-turn amplitude gives the otolith a longer path of motion.
(3) It does not result in the repositioning of the contralateral long arm lateral otolith.

Therefore, the 60° horizontal roll test can replace the supine roll test.

#### 3.5.2. The prone roll test

**Figure 7:**
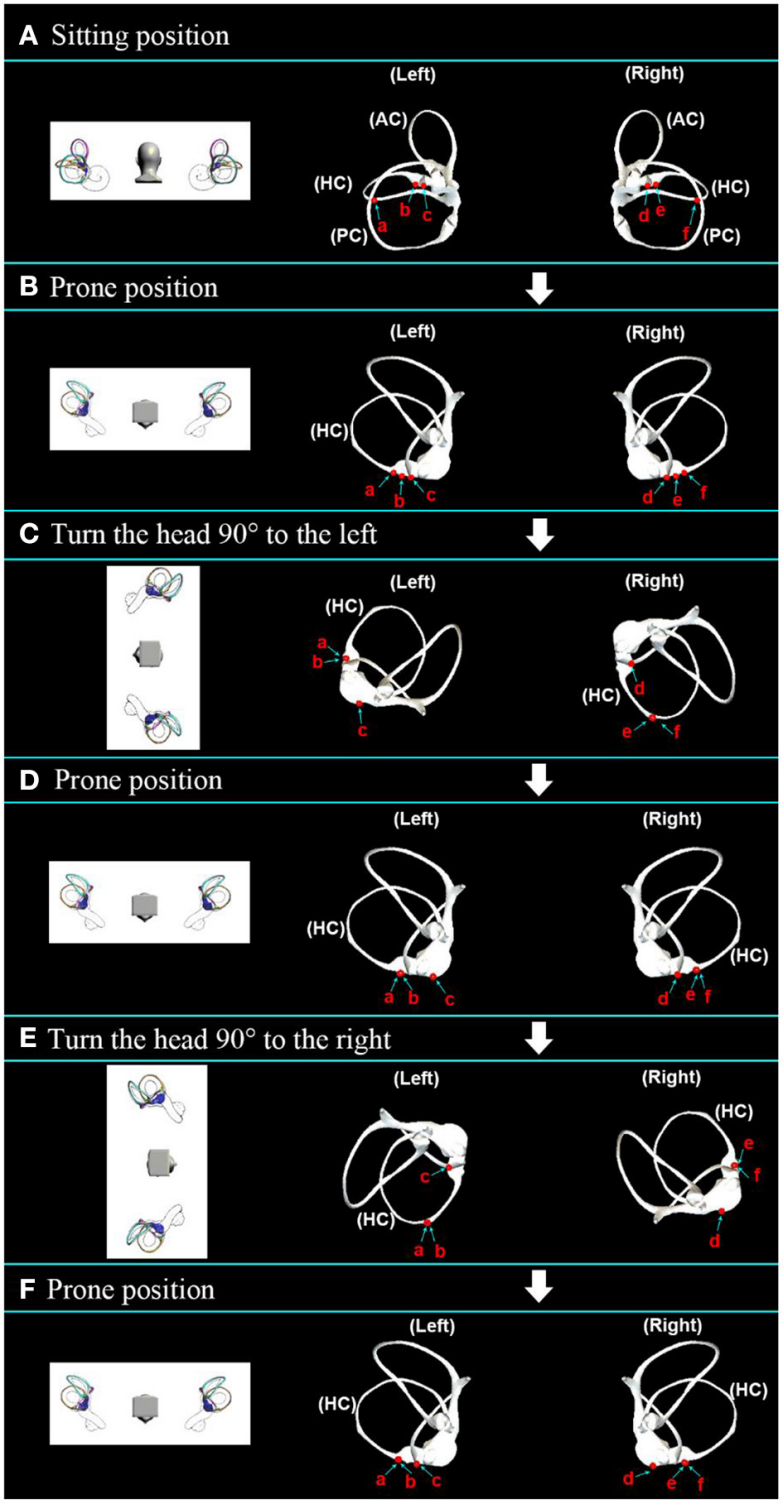
Movement of the otoliths in the horizontal semicircular canal in the prone roll test. **(A)** Initial distribution setting of the otoliths in the sitting position. **(B)** The stable position of the otoliths after transfer from the sitting position to the prone position. **(C)** The stable position of the otoliths after further rotation to the left by 90°. **(D)** The stable position of the otoliths after returning to the prone position. **(E)** The stable position of the otoliths after a further 90° rotation to the right. **(F)** The stable position of the otoliths after returning to the prone position.

The prone roll test was designed from the horizontal sitting position to the prone position, then turning the head 90° to the left, then turning 90° to the right to return to the prone position, then turning 90° to the right, and finally turn 90° to the left to return to the prone position. The prone roll test simulation was carried out and shown in Supplementary Video S5. The simulation results are shown in Figure 7 and Table 3, and it can be determined that the associated nystagmus caused by otolith movement is as follows:

(1) Step A: In the sitting position, the initial position of the otolith was set, as shown in Figure 7A.
(2) Step B: In the prone position, as shown in Figure 7B, the otolith movement behavior of the left and right ear semicircular canal is the same from the sitting position to the prone position. Taking the left side as an example, otolith a on the long arm side moved toward the ampulla, inducing horizontal left-facing nystagmus. Otolith b and c on the ampullary side of the long arm and the short arm moved to the bottom of the crista for a short distance. The cupulolithiasis was in an adhesion state.
(3) Step C: Turn the head 90° to the left, as shown in Figure 7C. In the left ear, otolith a and b on the long arm side remained at the bottom of the crista; otolith c on the short arm side moved toward the ampulla and entered the utricle, which induced apogeotropic nystagmus; the cupulolithiasis induced apogeotropic nystagmus. In the right ear, otolith e and f on the long arm side moved away from the ampulla to induce apogeotropic nystagmus; otolith d on the short arm side moved to the top of the crista in a short distance and induced apogeotropic nystagmus; the cupulolithiasis induced apogeotropic nystagmus.
(4) Step D: Turn the head 90° to the right to return to the prone position, as shown in Figure 7D. On the left side, the positions of otolith a, b, and c did not change significantly, and the cupulolithiasis was in an adhesion state. On the right side, otolith e and f on the side of the long arm moved toward the ampulla, causing horizontal right-facing nystagmus; otolith d moved to the bottom of the crista; the cupulolithiasis did not cause nystagmus.
(5) Step E: Turn the head 90° to the right, as shown in Figure 7E. On the left side, otolith a and b on the long arm moved away from the ampulla and induced apogeotropic nystagmus; otolith c moved in the utricle or moved away from the ampulla to the top of the crista to induce apogeotropic nystagmus; the cupulolithiasis induced apogeotropic nystagmus. On the right side, otolith e and f on the long arm moved to the bottom of the crista for a short distance; otolith d moved toward the ampulla to enter the utricle and induced apogeotropic nystagmus; the cupulolithiasis induced apogeotropic nystagmus.
(6) Step F: Turn the head 90° to the left to return to the prone position, as shown in Figure 7F. On the left, otolith a and b on the long arm moved toward the ampulla, inducing horizontal left-facing nystagmus; otolith c on the short arm moved in the utricle or moved to the bottom of the crista; the cupulolithiasis did not induce nystagmus. On the right side, the positions of otolith d, e, and f did not change significantly; the cupulolithiasis did not cause nystagmus.

**Table 3:** Otolith motion and nystagmus speculation in the prone roll test.

**Step A**	**Step B**	**Step C**	**Step D**	**Step E**	**Step F**
Otolith a was located on the PA of the LA	APM/←	The BC on the LA/–	The BC on the LA/–	AFM/weak ↑	APM/strong ←
Otolith b was located on the ampulla of the LA	The BC on the LA/–	The BC on the LA/–	The BC on the LA/–	AFM/weak ↑	APM/strong ←
Otolith c was located on the ampulla of the SA	The BC on the SA/–	APM into the utricle/strong ↑	Movement within the utricle/–	The TC on the SA/weak ↑	The BC on the SA/–
Otolith adhered to the cupula of the left ear	Adhesion state/–	Adhesion state/strong ↑	Adhesion state/–	Adhesion state/weak ↑	Adhesion state/–
Otolith d was located on the ampulla of the SA	The BC on the SA/–	The TC on the SA/weak ↑	The BC on the SA/–	APM into the utricle/strong ↑	Movement within the utricle/–
Otolith e was located on the ampulla of the LA	The BC on the LA/–	AFM/weak ↑	APM/ →	The BC on the SA/–	Short distance movement in the ampulla/–
Otolith f was located on the PA of the LA	APM/ →	AFM/weak ↑	APM/ →	The BC on the LA/–	Short distance movement in the ampulla/–
Otolith adhered to the cupula of the right ear	Adhesion state/–	Adhesion state/weak ↑	Adhesion state/–	Adhesion state/strong ↑	Adhesion state/–

–, Ineffective nystagmus; ↓, Geotropic nystagmus; ↑, Apogeotropic nystagmus; ←, Nystagmus horizontally toward the left side; →, Nystagmus horizontally toward the right side.

According to the characteristics of nystagmus induced by the prone roll test, the location of the otolith can be determined. In steps C and E, when only one nystagmus occurs, the otolith is considered to be on the long arm side, and nystagmus is induced when the head is turned to the uninvolved side. In addition, nystagmus that is evoked twice and lasts more than 1 min is considered to be cupulolithiasis, whereas otoliths are considered to be located on the short arm if the duration is <1 min. The nystagmus is more intense when the head is turned to the affected side.

Compared with the 90° supine roll test, the prone roll test has the following advantages:

(1) Added the latency period characteristic of nystagmus. The otolith of the horizontal semicircular canal moved to the bottom of the crista under the action of gravity, making the latency period a feature of nystagmus.
(2) It is easier to distinguish between canalolithiasis and cupulolithiasis. According to the number of nystagmuses induced by the prone roll test in the prone position, the difference between canalolithiasis and cupulolithiasis is more obvious without judging the intensity of nystagmus.
(3) The characteristics of nystagmus induced by repeated diagnostic tests are stable. Since the opening of the long arm side of the horizontal semicircular canal in the prone position is located at a high place, otoliths of the utricle will not enter the horizontal semicircular canal, and otoliths on the long arm side will not enter the utricle. The initial position of otolith movement is always the same, so the characteristics of nystagmus induced by repeated prone roll tests are always stable and consistent.

## 4. Discussion

BPPV virtual simulation is a useful tool for studying the diagnosis and treatment of BPPV. The critical point of applying a virtual simulation tool is establishing the membrane labyrinth model in a standard spatial coordinate system. Different spatial models of the semicircular canal may lead to inconsistent research results. For example, Bhandari's simulation of the Yacovino maneuver showed that the otolith located in the superior semicircular canal moved away from the ampulla (17). However, our previous research showed that the supine hanging head position could not extrude otoliths from the ampulla of the long arm of the superior semicircular canal. The main reason is that the membrane labyrinth model used by Bhandari was not accurate enough and did not take into account the local distension of the ampulla. Based on clinical microscopic CT data, we established a BPPV simulation model that was likely to the actual situation. We further measured the spatial attitude information of the semicircular canal and crista ampullaris.

The current preferred supine roll test for the diagnosis of HSC-BPPV has design flaws, such as turning over to the healthy side can lead to a repositioning of the otolith. In addition, in clinical practice, the direction and intensity of nystagmus induced by repeated tests may also change, affecting the sensitivity of diagnostic tests (25). The 180° supine roll test was more likely to induce vertigo and nystagmus than the 90° supine roll test. However, it does not correct the design flaw of otolith repositioning. Martellucci proposed a new paradigm for the diagnosis of HSC-BPPV in the upright position, which provides more sensitive diagnostic indicators and avoids the discomfort associated with the supine position. However, there are limitations in distinguishing between canalolithiasis and cupulolithiasis (12).

We have developed the 60° roll test, which effectively corrected some defects of the supine roll test by reducing the amplitude of turning the head to one side. During diagnosis, this does not result in repositioning of the otolith on the long arm. In addition, the 60° roll test is easy to operate without turning over, which can also avoid cervical spine injury. More importantly, with a head turn of 120°, the path of otolith motion in the long arm of the HSC is longer than 90°. This results in a stronger hydrodynamic effect and higher sensitivity.

In addition, we also designed the prone roll test. In the prone position, the opening on the long arm side of the horizontal semicircular canal was higher. In this way, the long-arm otoliths cannot enter the utricle, or the utricle otoliths cannot enter the duct. Moreover, otoliths on the long arm side were concentrated in the ampulla of the long arm side. This means that the starting position of the otolith movement is always the same. Therefore, the characteristics of nystagmus induced by repeated diagnostic tests are more stable and consistent. Moreover, otoliths of the horizontal semicircular canal slide to the bottom of the crista ampullaris under gravity, adding to the character of the nystagmus latency period. It can be more convenient for the physician to make a diagnosis. More importantly, instead of distinguishing between canalolithiasis and cupulolithiasis by comparing the strength of the nystagmus, the location of the otolith can be located by the nystagmus induced by the different steps of the maneuver, which is not only more convenient and easier, but also significantly improves the fault tolerance rate of diagnosis.

When it is necessary to repeat the diagnostic test to observe the nystagmus features to further confirm the diagnosis of BPPV. To avoid repeatedly inducing vertigo during the repositioning process, we suggest that the repositioning method can start in the prone position, with the healthy ear down, then return to the prone position, then the affected ear down, continue turning over to the healthy side of the body, then continue turning the head 45° to 90° and sitting up.

In canalolithiasis, the healthy ear first downward does not induce vertigo, so the patient does not feel pain. The critical step for cupulolithiasis is to make the otolith attached to the crista ampullaris fall off and convert it into canalolithiasis. Crista-cap otoliths can be located on the side of the short arm and the side of the long arm. The recumbent position on the healthy ear side can promote crista-cap otoliths on the short arm side to fall off and induce intense vertigo and nystagmus. The recumbent position on the affected ear side can cause crista-cap otoliths on the long arm side to fall off and induce weak vertigo and nystagmus. As otoliths on the short arm of the horizontal semicircular canal are prone to spontaneous reduction, otoliths on the long arm are more common. Therefore, it is more reasonable to give priority to the recumbent position on the affected side.

However, whether turning to the affected ear side decubitus position or the healthy ear side decubitus position may induce vertigo and nystagmus, which many patients cannot tolerate.

To alleviate the discomfort of the patient, it can be considered to adopt the prone position for a long time or the prone position with the head bowed at 16°. In this position, the crista ampullaris of the horizontal semicircular canal is parallel to the direction of gravity, and the crista-cap is in a high position, which can make the otolith attached to the top of the crista ampullaris fall off. If the crista-cap otolith is still difficult to fall off, the patient may further try kowtowing or sleeping on his stomach. These two methods have significant effects on the conversion of the crista-cap otolith to the duct otolith on the short and long arm sides.

If a repeated prone roll test no longer induces vertigo and nystagmus with the head turned to the healthy ear, this indicates that the cupulolithiasis has transformed into canalolithiasis. When the head is turned to the affected ear side without nystagmus, the otolith has been returned to the utricle. It is considered that the initial condition is cupulolithiasis on the short arm side, which is converted into canalolithiasis and returned to the utricle when the head rotates to the healthy ear side.

## 5. Conclusion

In conclusion, both the 90° supine roll test and the 180° supine roll test have the disadvantage of repositioning the otoliths, which reduces the sensitivity of the diagnostic test. The 60° roll test is primarily designed to correct this deficiency and can replace these two tests. However, these three tests still have a shortcoming in that the otoliths in the utricle can enter the semicircular canal in the supine position. The prone roll test can solve this problem and is suitable for checking the diagnostic test after repositioning, which is safer. And not only can it effectively distinguish canalolithiasis from cupulolithiasis, but it also makes it easier to judge the location of the otolith, and the induced nystagmus features are more clear and consistent. In cupulolithiasis, the continuous prone position can also promote the conversion of cupulolithiasis into canalolithiasis, thus distinguishing short-arm-side cupulolithiasis from long-arm-side cupulolithiasis. We also performed clinical validation of the new diagnostic maneuvers, especially the 60° roll test, in more than 1 year of clinical practice. Patients with negative results on the 60° roll test were not found to be positive on further examination of the 90° supine roll test. It is necessary to carry out reasonable design and implementation of clinical verification tests of 60° roll test and supine roll test in the future.

## Data availability statement

The original contributions presented in the study are included in the article/Supplementary material, further inquiries can be directed to the corresponding author.

## Author contributions

YL: conceptualization, methodology, investigation, formal analysis, and writing—original draft. XY: conceptualization, methodology, funding acquisition, resources, supervision, and writing—review and editing. All authors contributed to the article and approved the submitted version.

## References

[1] Pagnini P Nuti D Vannucchi P. Benign paroxysmal vertigo of the horizontal canal. ORL. (1989) 51:161–70. 10.1159/000276052 2734007

[2] Squires TM Weidman MS Hain TC Stone HA. A mathematical model for top-shelf vertigo: the role of sedimenting otoconia in BPPV. J Biomech. (2004) 37:1137–46. 10.1016/j.jbiomech.2003.12.014 15212918

[3] Hall SF Ruby RR McClure JA. The mechanics of benign paroxysmal vertigo. J Otolaryngol. (1979) 8:151–8. 430582

[4] Schuknecht HF. Cupulolithiasis. Arch Otolaryngol. (1969) 90:765–78. 10.1001/archotol.1969.00770030767020 5353084

[5] House MG Honrubia V. Theoretical models for the mechanisms of benign paroxysmal positional vertigo. Audiol Neurootol. (2003) 8:91–9. 10.1159/000068998 12634457

[6] Cakir BO Ercan I Cakir ZA Civelek S Sayin I Turgut S. What is the true incidence of horizontal semicircular canal benign paroxysmal positional vertigo? Otolaryngol Head Neck Surg. (2006) 134:451–4. 10.1016/j.otohns.2005.07.045 16500443

[7] Moon SY Kim JS Kim B-K Kin JI Lee H Son SI. Clinical characteristics of benign paroxysmal positional vertigo in Korea: a multicenter study. J Korean Med Sci. (2006) 21:539–43. 10.3346/jkms.2006.21.3.539 16778402 PMC2729964

[8] Baloh RW Honrubia V. Clinical neurophysiology of the vestibular system. Contemp Neurol Ser. (1979) 18:1–21. 378525

[9] Bashir K Yousuf A Rauf L Dewji M Elmoheen A. Curing Benign Paroxysmal Positional Vertigo (BPPV) through telehealth: a case series. Cureus. (2021) 13:e16363. 10.7759/cureus.16363 34395140 PMC8360323

[10] Zhang X Bai Y Chen T Wang W Han X Li S. A show of Ewald's Law: I Horizontal semicircular canal benign paroxysmal positional vertigo. Front Neurol. (2021) 12:632489. 10.3389/fneur.2021.632489 33613438 PMC7887281

[11] Bhattacharyya N Gubbels SP Schwartz SR Edlow JA El-Kashlan H Fife T. Clinical practice guideline: benign paroxysmal positional vertigo (update). Otolaryngol Head Neck Surg. (2017) 156:S1–47. 10.1177/0194599816689667 28248609

[12] Martellucci S Malara P Castellucci A Pecci R Giannoni B Marcelli V. Upright BPPV protocol: feasibility of a new diagnostic paradigm for lateral semicircular canal benign paroxysmal positional vertigo compared to standard diagnostic maneuvers. Front Neurol. (2020) 11:578305. 10.3389/fneur.2020.578305 33329319 PMC7711159

[13] Bhandari A Bhandari R Kingma H Zuma EMF Strupp M. Three-dimensional simulations of six treatment maneuvers for horizontal canal benign paroxysmal positional vertigo canalithiasis. Eur J Neurol. (2021) 28:4178–83. 10.1111/ene.15044 34339551

[14] Strupp M Goldschagg N Vinck AS Bayer O Vandenbroeck S Salerni L. BPPV: comparison of the SemontPLUS with the semont maneuver: a prospective randomized trial. Front Neurol. (2021) 12:652573. 10.3389/fneur.2021.652573 33935951 PMC8079727

[15] Kong L Wu J Feng M Zhang Y Liu Z Yang X. Virtual simulation of otolith movement for the diagnosis and treatment of benign paroxysmal positional vertigo. Biomed Tech. (2021) 66:387–93. 10.1515/bmt-2020-0278 33567178

[16] Peng Y Qi J. A dynamic model of otolith for determinants of a BPPV reposition maneuver with its simulation. J Phys Conf Ser. (2020) 1631:012044. 10.1088/1742-6596/1631/1/012044

[17] Bhandari A Kingma H Bhandari R. BPPV simulation: a powerful tool to understand and optimize the diagnostics and treatment of all possible variants of BPPV. Front Neurol. (2021) 12:632286. 10.3389/fneur.2021.632286 33841305 PMC8032929

[18] Zheng Y Wu S Yang X. Analysis of Dix-Hallpike maneuver induced nystagmus based on virtual simulation. Acta Otolaryngol. (2021) 141:433–9. 10.1080/00016489.2021.1876247 33557660

[19] Yang X Gao L. Simulation study of BPPV fatigability. Front Neurol. (2022) 13:874699. 10.3389/fneur.2022.874699 35599733 PMC9121120

[20] Wu S Li J Zhou M Yang X. Simulation study of canal switching in BPPV. Front Neurol. (2022) 13:944703. 10.3389/fneur.2022.944703 35911897 PMC9326062

[21] Wu S Lin P Zheng Y Zhou Y Liu Z Yang X. Measurement of human semicircular canal spatial attitude. Front Neurol. (2021) 12:741948. 10.3389/fneur.2021.741948 34630312 PMC8498035

[22] David R Stoessel A Berthoz A Spoor F Bennequin D. Assessing morphology and function of the semicircular duct system: introducing new *in-situ* visualization and software toolbox. Sci Rep. (2016) 6:32772. 10.1038/srep32772 27604473 PMC5015051

[23] Scarpa A Cassandro C Gioacchini FM Viola P Cuofano R Kaleci S. Lateralization of horizontal semicircular canal benign paroxysmal positional vertigo (HSC-BPPV) with the latency test: a pilot study. Acta Otolaryngol. (2019) 139:854–9. 10.1080/00016489.2019.1635712 31282790

[24] Sekine K Imai T Nakamae K Miura K Fujioka H Takeda N. Dynamics of the vestibulo-ocular reflex in patients with the horizontal semicircular canal variant of benign paroxysmal positional vertigo. Acta Otolaryngol. (2004) 124:587–94. 10.1080/00016480310002131 15267177

[25] Lee SH Kim MK Cho KH Kim JS. Reversal of initial positioning nystagmus in benign paroxysmal positional vertigo involving the horizontal canal. Ann N Y Acad Sci. (2009) 1164:406–8. 10.1111/j.1749-6632.2008.03739.x 19645938

